# Diagnostic and management of life-threatening Adult-Onset Still Disease: a French nationwide multicenter study and systematic literature review

**DOI:** 10.1186/s13054-018-2012-2

**Published:** 2018-04-11

**Authors:** Antoine Néel, Anaïs Wahbi, Benoit Tessoulin, Julien Boileau, Dorothée Carpentier, Olivier Decaux, Laurence Fardet, Guillaume Geri, Pascal Godmer, Cécile Goujard, Hervé Maisonneuve, Arnaud Mari, Jacques Pouchot, Jean-Marc Ziza, Cédric Bretonnière, Mohamed Hamidou

**Affiliations:** 1 Service de Médecine Interne, PHU3, CHU Hôtel-Dieu, 44093 Nantes, France; 2 Service d’Hématologie, PHU1, CHU Hôtel-Dieu, 44093 Nantes, France; 3 Service de Médecine, CH de Morlaix, 29672 Morlaix, France; 4 Service de Réanimation Médicale, CHU de Rouen, 76031 Rouen, France; 5 Service de Médecine Interne, CHU de Rennes, 35033 Rennes, France; 6 Service de Dermatologie, Hôpital Henri Mondor, 94000 Créteil, France; 7 Service de Réanimation Médicale, CHU Cochin, AP-HP, 75012 Paris, France; 8 CH Bretagne-Atlantique, 56000 Vannes, France; 9 Service de Médecine Interne, CHU Bicêtre, AP-HP, 94270 Kremlin-Bicêtre, France; 10 Service de Médecine Interne, CHD Vendée, 85925 La Roche-sur-Yon, France; 11 Service de Réanimation, Hôpital Yves Le Foll, 22000 St Brieuc, France; 12 Service de Médecine Interne, Hôpital Européen Georges Pompidou, AP-HP, 75908 Paris, France; 13 Service de Médecine Interne-Rhumatologie, groupe hospitalier Diaconesses-Croix-Saint-Simon, 75020 Paris, France; 14 Service de Réanimation Médicale, PHU3, CHU de Nantes, 44093 Nantes, France; 15 UPRES EA 3826, Faculté de Médecine, Université de Nantes, 44035 Nantes, France

**Keywords:** Adult onset Still disease, Reactive hemophagocytic syndrome, Shock, Differential diagnosis, Anakinra, Cyclosporin, Intravenous immunoglobulins, ICU

## Abstract

**Background:**

Adult-onset Still disease (AOSD) is a rare systemic inflammatory disorder. A few patients develop organ complications that can be life-threatening. Our objectives were to describe the disease course and phenotype of life-threatening AOSD, including response to therapy and long-term outcome.

**Methods:**

A multicenter case series of intensive care medicine (ICU) patients with life-threatening AOSD and a systematic literature review.

**Results:**

Twenty patients were included. ICU admission mostly occurred at disease onset (90%). Disease manifestations included fever (100%), sore throat (65%), skin rash (65%), and arthromyalgia (55%). Serum ferritin was markedly high (median: 29,110 ng/mL). Acute respiratory failure, shock and multiple organ failure occurred in 15 (75%), 10 (50%), and 7 (35%) cases, respectively. Hemophagocytosis was demonstrated in eight cases. Two patients died. Treatment delay was significant. All patients received corticosteroids. Response rate was 50%. As second-line, intravenous immunoglobulins were ineffective. Anakinra was highly effective*.* After ICU discharge, most patients required additional treatment*.* Literature analysis included 79 cases of AOSD with organ manifestations, which mainly included reactive hemophagocytic syndrome (42%), acute respiratory failure (34%), and cardiac complications (23%). Response rate to corticosteroids was 68%. Response rates to IVIgs, cyclosporin, and anakinra were 50%, 80%, and 100%, respectively.

**Conclusions:**

AOSD should be recognized as a rare cause of sepsis mimic in patients with fever of unknown origin admitted to the ICU. The diagnosis relies on a few simple clinical clues. Early intensive treatment may be discussed. IVIgs should be abandoned. Long-term prognosis is favorable.

**Electronic supplementary material:**

The online version of this article (10.1186/s13054-018-2012-2) contains supplementary material, which is available to authorized users.

## Background

Adult-onset Still disease (AOSD) is a rare systemic inflammatory disorder of unknown etiology. Its prevalence is less than 1/100,000 and it affects predominantly young people [1]. AOSD typically presents with high-grade fever, evanescent rash, sore throat, arthromyalgia, arthritis, serositis, discrete lymphadenopathy, hepatosplenomegaly, neutrophilic leukocytosis, hepatic cytolysis, and high serum ferritin [2, 3].

While differential diagnosis is broad (infection, malignancy, autoimmunity) there is no single biological nor pathological finding specific for AOSD [2]. Thus it is an experience-based diagnosis. The long-term course may be monocyclic, polycyclic, and/or complicated by a chronic erosive polyarthritis [2]. AOSD treatment relies on corticosteroids (CS) and immunosuppressive agents such as methotrexate or cyclosporin [4–6]. Recently, biologics (off-label) targeting tumor necrosis factor alpha (TNF-α) [7–10] and, more importantly, interleukin (IL)-1 (anakinra) [11–14] or IL-6 (tocilizumab) [7, 14, 15] have shown interesting efficacy in refractory chronic AOSD.

A few patients develop organ complications that can be life-threatening, including reactive hemophagocytic syndrome (RHS), cardiac failure, respiratory distress, coagulopathy, severe hepatitis, and multiple organ failure (MOF) [16]. Data regarding these infrequent complications mainly arise from case reports or small series focusing on one manifestation such as RHS [17–19] or myocarditis [20]. Despite these patients posing important diagnostic and therapeutic dilemmas, little is known about life-threatening AOSD as a whole.

Our objectives were: (i) to describe the disease course and phenotype of life-threatening AOSD (i.e. cases with organ failure leading to ICU admission) (ii) to analyze the response to therapy and long-term outcome of these patients.

We report a multicenter case series of 20 intensive care unit (ICU) patients with life-threatening AOSD as well as a systematic literature review of organ manifestation of AOSD.

## Methods

### Multicenter case series

#### Inclusion criteria

This multicenter retrospective study was performed under the auspice of the *French Intensive Care Society* and the *French Internal Medicine Society*. Inclusion criteria were: (i) admission to ICU due to AOSD-related organ failure. (ii) AOSD diagnosis fulfilling the Yamaguchi [21] and/or Fautrel [22] criteria. (ii) Exclusion of differential diagnosis, including infection, malignancy, and other systemic immune mediated disorders. (iv) Age at AOSD diagnosis > 18 years. (v) Organ failure requiring organ-supporting therapeutic intervention including vasopressor agents, pericardial drainage, mechanical ventilation, renal replacement therapy (RRT) or plasmatherapy. Exclusion criteria were ICU admission without organ failure or for reasons other than AOSD.

The flow chart is in Additional file 1. This observational study was performed in accordance with the Helsinki declaration, and European and French ethics laws.

#### Data collection

Data were collected using a standardized form by one of the investigators (AW). Disease severity was measured using the SAPS II (Simplified Acute Physiology Score II). Organ failure definitions were adapted from SAPS II and Logistic Organ Dysfunction (LOD) system [23–25]. Definitions of respiratory and cardio-circulatory failures, acute kidney injury (AKI), neurologic dysfunction, and hematologic disorders are reported in Additional file 2. Response to therapy was defined as ICU discharge.

### Systematic literature review

Using MEDLINE via PubMed (National Library of Medicine, Bethesda, MD, USA), we performed a computer search for English or French language publications from January 2000 to March 2014. We selected case reports from western or east Asian countries describing AOSD cases complicated by RHS or/and shock, respiratory distress, myocarditis, tamponade, hepatic failure, thrombotic microangiopathy (TMA), disseminated intravascular coagulation (DIC), MOF, AKI, and/or neurological involvement. Individual data were collected by one of the investigators (AW). Details of systematic literature review are available in Additional file 2.

### Statistical analysis

Data are presented as median (min-max). Analyses were performed using SPSS software, version 21.0 (IBM Corp, Armonk, NY, USA). Continuous variables were compared using the nonparametric Mann-Whitney test. Statistical significance was defined as a two-tailed *p* value of < 0.05. Cumulative incidence and survival curve were created using the Kaplan-Meier method (GraphPad Prism version 5 (GraphPad Software, San Diego, CA, USA). Venn diagrams were drawn using the online tool available at http://bioinformatics.psb.ugent.be/webtools/Venn/.

## Results

### Clinical and biological AOSD features

**Fig. 1:**
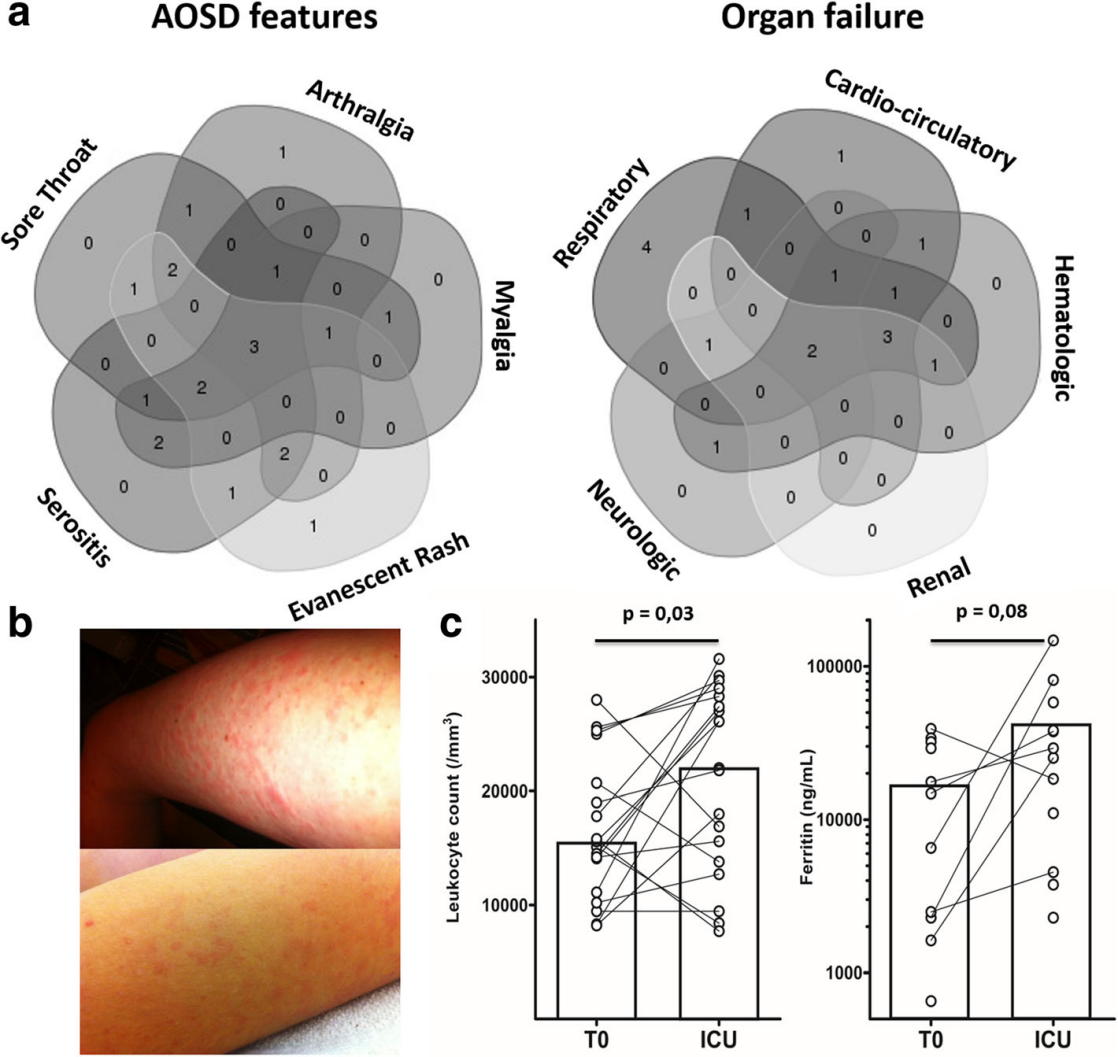
Phenotypic spectrum of life-threatening AOSD. **a** Venn diagram depicting the association and overlap of five key clinical features of AOSD with organ failure. **b** Evanescent skin rash in two patients with AOSD. **c** Kinetics of leukocytosis and ferritinemia. *T0* before ICU admission, *ICU* first recorded value in the ICU

Twenty patients (8 women and 12 men) from 11 centers were included. Patients’ ethnicity was mostly Caucasian (18/20, 80%). Median age was 36 years (18–64). ICU admission mostly occurred during the initial AOSD flare (90%). Patients exhibited a combination of fever, evanescent skin rash, sore throat, arthromyalgia, serositis (Fig. 1a and b), and organ involvement (Table 1). Eighteen patients (90%) had leukocytosis ≥10 × 10^9^/L (median: 22 × 10^9^/L). All patients had marked biological inflammation (median C-reactive protein [CRP] level: 328 mg/L (100–495)) contrasting with fibrinogen levels (median: 3.8 g/L (1.1–7.1)). Prothrombin activity (PA, normal range: 70–100%) was ≤50% in eight cases (40%). Ten patients (50%) had elevated liver enzymes (median aspartate aminotransferase [ASAT] level: 85.5 UI/L (31–2963)). Serum ferritin was markedly elevated (median: 29110 ng/mL (2287–554,944)) (Fig. 1c).

**Table 1:** Characteristics of life-threatening AOSD

	*n* (%)
Characteristics of patients	
Age, years (median)	36 (18–64)
Male	12 (60%)
First episode	18 (90%)
AOSD features	
Fever	20 (100%)
Rash	13 (65%)
Sore throat	13 (65%)
Arthromyalgia	17 (85%)
Arthritis	3 (15%)
Hepatosplenomegaly	13 (65%)
Organ manifestations	
Respiratory	
Acute respiratory failure	15 (75%)
Lung infiltrate	12 (60%)
Pleural effusion	12 (60%)
Cardiocirculatory	
Shock	10 (50%)
Myocarditis	8 (40)
Pericarditis	10 (50%)
Tamponade	3 (16%)
Hematologic	
Disseminated intravascular coagulation	10 (50%)
Hemophagocytosis	8 (40%)
Thrombotic microangiopathy	1 (5%)
Other	
Acute kidney injury	7 (35%)
Neurologic dysfunction	5 (25%)
Severe hepatitis	3 (15%)
Multiple organ failure	8 (40%)
Death	2 (10%)

### Disease course and diagnostic delay

**Fig. 2:**
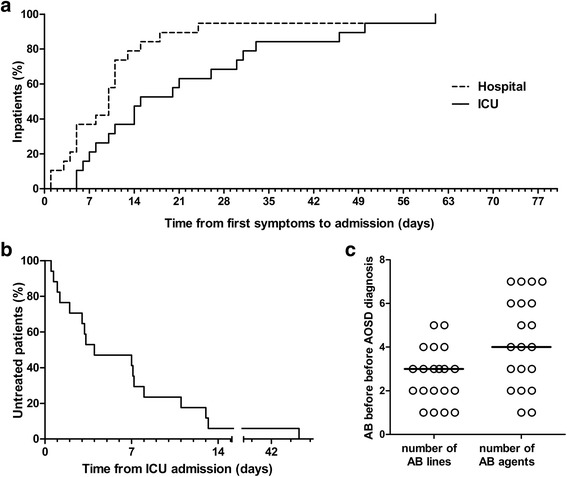
Disease course prior to ICU admission and therapeutic delay. **a** Time from first symptoms (i.e. fever onset) to hospitalization and to ICU admission. **b** Treatment delay among 17 untreated patients upon ICU admission. **c** Antibiotic use per patient, prior to AOSD recognition

Median time between fever onset and hospitalization was 10 days (1–61) (Fig. 2a). Median time between hospitalization and ICU admission was 4 days (0–39). Median time between fever onset and ICU admission was 14.5 days (5–61). Most patients (17/20, 85%) had received no specific treatment for AOSD upon ICU admission. Even then, treatment began more than 2 days after ICU admission in most cases (12/17, 60%) (Fig. 2b). One patient was treated after 44 days spent in the ICU. Suspected diagnoses included septic shock, pneumonia, angiocholitis, Lemierre syndrome, Rickettsiosis, Leptospirosis, drug reaction. As shown in Fig. 2c, many antibiotics were prescribed before AOSD was eventually considered.

### Organ involvement

Organ involvement mainly affected the respiratory, cardiocirculatory and hematologic systems (Fig. 1a). The median SAPS II score was 33 (16–88).

#### Pulmonary involvement

Fifteen patients (75%) suffered acute respiratory distress (ARF) requiring mechanical ventilation (invasive in 11, noninvasive in 4). One patient required pleural drainage. Upon chest computed tomography (CT), 12 patients (60%) exhibited parenchymal lesions, including pulmonary condensations (35%), bilateral micronodular infiltrates (15%) and/or interstitial pneumonia (10%). Pleural effusions were frequent (12, 60%). Respiratory distress fulfilled the acute respiratory distress syndrome (ARDS) criteria in two patients, who were treated by prone position and neuromuscular blocking agents. Four patients had tracheostomy for weaning from mechanical ventilation (20%).

#### Cardiac and circulatory involvement

Ten patients had shock requiring vasopressors. Five had non-cardiogenic shock, with MOF in four cases. Norepinephrine was administered for a median of 8 days (2–15). Four other patients had cardiogenic shock requiring dobutamine for a median of 3 days (2–8) and one patient had mixed shock. One patient needed an extracorporeal life support system for 7 days.

Pericarditis occurred in ten patients (50%) with cardiac tamponade in three (drainage in two cases). Five patients (25%) had < 45% left ventricular ejection fraction. Their median was 40 (25–45). No patients had ventricular heart rhythm disorder. Eight patients had evidence of myocarditis (40%), which was occasionally proven by magnetic resonance imaging (MRI) or endomyocardial biopsy (one case each).

#### Hematologic disorders

**Fig. 3:**
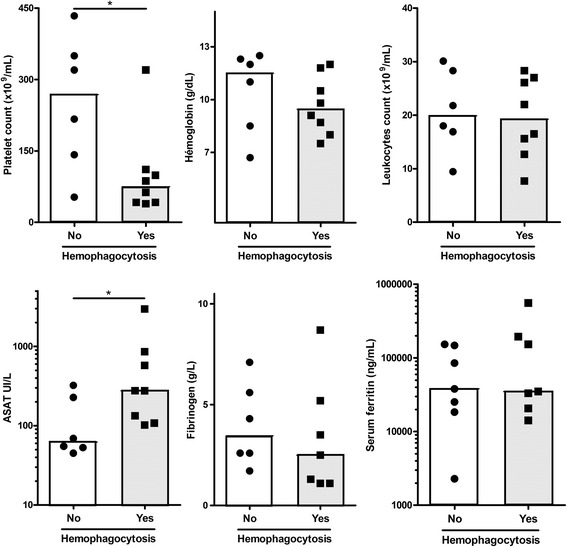
Biological correlates of bone marrow hemophagocytosis. Fourteen patients have had bone marrow examination. Key biological parameters are compared according to the presence or absence of hemophagocytosis in the bone marrow. Histograms depict the median. *p < 0.05, Mann-Whitney test

Nineteen patients had moderate anemia. None had leucopenia. Seven patients had platelet count < 100 × 10^9^/L. Fourteen patients underwent bone marrow examination. Hemophagocytosis was seen in eight cases and correlated with thrombocytopenia and liver enzyme elevation. In contrast, hemoglobin, leukocyte, fibrinogen and ferritin levels did not predict hemophagocytosis (Fig. 3).

Ten patients (50%) had DIC in the context of MOF (*n* = 6), severe hepatitis (*n* = 2), myocarditis (*n* = 1) or ARF (*n* = 1). One patient had TMA (5%), with thrombocytopenia, hemolytic anemia, and schizocytosis. TMA was confirmed by renal biopsy and it was complicated by AKI requiring RRT, and status epilepticus. A disintegrin and metalloprotease with thrombospondin type I repeats-13 (ADAMTS 13) activity was 35%.

#### Other

Liver enzyme elevation above 3 × ULN occurred in ten patients (50%). One patient had hepatic encephalopathy and two had hepatic failure with PA < 50%. None received a liver transplant. Neurological impairment occurred in five patients (25%), in association with other organ failure. AKI occurred in seven patients (35%, median creatinine level: 337 μmol/L (233–580)). All seven patients required RRT (median duration = 8 days (4–30)). AKI occurred in the event of MOF for six patients (85,7%) and TMA for one (14.3%). MOF occurred in seven patients (35%), with hemophagocytosis for five patients.

### Treatment and outcomes

#### Treatment efficacy in the ICU

As first line, all patients received high-dose CS. Response to CS monotherapy, i.e. leaving the ICU without further treatment, was achieved in only ten cases (50%), within a median of 6 days (1–36). These patients had lower ferritin level (*p* = 0,017) and tended to exhibit a less severe clinical picture. An additional table shows this in more details (see Additional file 3).

In the ten remaining patients, a second-line treatment was added after several days of CS. Detailed individual data are available in Additional file 4. Most frequently used second-line treatment was intravenous immunoglobulins (IVIgs) at 2 g/kg/day (6/10, 60%). Treatment efficacy is summarized in Table 3. IVIgs were ineffective. Anakinra was eventually used in five cases at 100 mg/day and was highly effective. An additional figure shows this in more details (see Additional file 5). Median time until response to anakinra was 4.5 days (3–15).

#### ICU complications

Median ICU stay duration was 13 days (2–82). Ten patients (50%) suffered at least one complication, including infection, hemorrhage, distal necrosis, and ICU neuropathy (Table 2).

**Table 2:** ICU complications

ICU complications	*n* = 20
Infection	7 (35%)
Hemorrhage	6 (30%)
Hypertensive emergency	1 (5%)
Atrial fibrillation^a^	3 (15%)
Hypoxic cardiocirculatory arrest^b^	1 (5%)
Distal extremity necrosis ^c^	2 (10%)
ICU neuropathy	3 (15%)
Tracheotomy	4 (20%)
Death	2 (10%)

*ICU* intensive care unit, ^ a^with pulmonary edemae, ^b^full recovery, ^c^Caused by disseminated intravascular coagulation for one, and thrombotic microangiopathy for the other

Seven patients developed ten infectious complications. Six patients (30%) developed catheter-related infection, with suppurative thrombophlebitis in two cases. Causal agents included *Staphylococci* (*n* = 3), *Pseudomonas aeruginosa* (*n* = 3), *Corynebacterium jekeium* (*n* = 1), *Candida albicans* (*n* = 1). Ventilator-associated pneumonia occurred in two patients (5%), caused by *Pseudomonas aeruginosa* plus *Klebsiella pneumonia* in one, *Enterobacter aerogenes* in the other.

Six patients (30%) experienced hemorrhages in the context of hemostasis disorders, thrombocytopenia and/or organ lesions: digestive hemorrhage by angiodysplasia (*n* = 1), peptic ulcer (*n* = 1), and intestinal necrosis (*n* = 1); liver biopsy (*n* = 1); lower limb (*n* = 1); and diffuse hemorrhage (*n* = 1). Distal extremity necrosis occurred in two patients (10%), caused by DIC for one, and TMA for the other. Three patients (15%) had atrial fibrillation, complicated by pulmonary edema. One patient had hypoxic cardiocirculatory arrest, with full recovery. One patient had hypertensive emergency. Three patients (15%) developed ICU neuropathy. Four patients (20%) needed tracheotomy.

Two patients (10%) died in the ICU. Both had MOF and hemophagocytosis. A 19-year-old man, with a previous history of AOSD died after ineffective CS then IVIgs treatment. Treatment had begun 17 days after first signs. A 37-year-old woman with new-onset AOSD died after 2 months in the ICU. This patient presented with hemophagocytosis uncontrolled by CS, anakinra, cyclosporine, and etoposide. She underwent diagnostic splenectomy and suffered repeated infections, notably invasive candidiasis. Autopsy disclosed hepatosplenic hemophagocytosis. Treatment had begun 32 days after fever onset.

#### Long-term outcome

After leaving the ICU, 11 patients with persistent disease activity required additional treatment before discharge. At 3 months, all patients were on CS and 16/18 (89%) received additional treatment, mostly anakinra (*n* = 10) and/or methotrexate (*n* = 5). Detailed long-term treatment data are reported in Table 3. At last follow-up, a single patient had received no other treatment than CS. The clinical course of AOSD was monocyclic, polycyclic and articular in ten (56%), six (33%), and two patients (11%), respectively. After a median follow-up of 3.5 years, no patient had suffered life-threatening relapse.

**Table 3:** Initial treatment efficacy and long-term therapeutic data

	Initial treatment efficacy		Treatment received after leaving the ICU		
	*n* = 20		*n* = 18		
			At 3 months	At 1 year	At 2 years
Treatment	n	Efficacy	*n* (%)	*n* (%)	*n* (%)
Corticosteroids alone	20	10 (50%)	4/18 (22%)	3/18 (17%)	3/13 (23%)
IVIgs	6	1 (17%)	1/18 (5%)	0	0
Cyclosporine	3	1 (33%)	3/18 (17%)	2/18 (11%)	0
Anakinra (IL1Ra)	5	4 (80%)	10/18 (56%)	7/18 (39%)	3/13 (23%)
Etoposide	3	2 (67%)	0	0	0
Methotrexate			5/18 (28%)	5/18 (28%)	4/13 (31%)
Leflunomide			1/18 (5%)	0	0
TNF-α blockers			1/18 (5%)	1/18 (5%)	1/13 (7%)
Cyclophosphamide			0	1/18 (5%)	1/13 (7%)
Azathioprine			0	1/18 (5%)	1/13 (7%)
Tocilizumab (anti-IL6)			0	1/18 (5%)	0
Remission off treatment			0	2/18 (5%)	3/13 (23%)

*ICU* intensive care unit, *IL1Ra* interleukin-1 receptor antagonist, *IVIgs* intravenous immunoglobulins, *TNF* tumour necrosis factor

### Systematic literature review

We performed a systematic review of published data describing AOSD patients with organ complications, which included 79 cases from 62 publications. Median age at life-threatening episode was 38 years. Fifty patients were female (63.3%). Organ complication mostly occurred at diagnosis (*n* = 62, 78%). The spectrum of organ complications were as follows: 33 RHS (42%)*;* 27 ARF (34%) including six ARDS (8%); 18 cardiac complications (23%) including six tamponades (8%), 12 myocarditis (15%), complicated by cardiogenic shock in five cases (6%); 16 MOF (20.3%); 13 TMA (16.5%); four fulminant hepatitis (5.1%) with two liver transplantations. As in our series, neurologic and renal impairment resulted from other organ manifestations.

As first line, most patients received CS monotherapy (75/79, 95%). Response rate was 68%. The efficacy of second-line treatments is reported in Table 4. When data from our series and literature review were pooled, the efficacy rate of IVIgs, cyclosporine, and anakinra were 11/27 (41%), 13/18 (72%), and 8/9 (89%), respectively (Table 4).

**Table 4:** Initial treatment efficacy and long-term therapeutic data in ICU

	Initial treatment efficacy				
	Literature (*n* = 79)		Present series (*n* = 20)		Pooled
Treatment	*n*	Efficacy	*n*	Efficacy	Efficacy
Corticosteroids alone	75	51 (68%)	20	10 (50%)	61/95 (64%)
IVIg	21	10 (48%)^a^	6	1 (17%)	11/27 (41%)
Cyclosporine	15	12 (80%)	3	1 (33%)	13/18 (72%)
Anakinra (IL1Ra)	4	4 (100%)	5	4 (80%)	8/9 (89%)
Etoposide	0	0 (0%)	3	2 (67%)	2/3 (67%)

*ICU* intensive care unit, *IL1Ra* interleukin1 receptor antagonist, *IVIg* intravenous immunoglobulins, ^a﻿^Efficacy of IVIg monotherapy = 5/9 (55.6%)

## Discussion

Herein, we report on the first study focusing on AOSD patients requiring ICU admission due to AOSD-related organ failure. Our objectives were to describe the phenotypic spectrum, response to therapy and long-term outcome of this infrequent subgroup of patients with life-threatening AOSD.

Until now, most studies about AOSD have focused on the long-term risk of developing further systemic flare(s) and/or a chronic polyarthritis. Similarly, most data regarding therapeutics focuses on patients with refractory, chronic, and non-life-threatening disease [1–15, 21, 22]. The fact that AOSD patients may develop severe organ manifestations is mentioned for decades, but this subset of patients has been seldom studied [2]. The present work is the first attempt to study critically ill AOSD patients as a whole. The frequency of organ complications is difficult to estimate, but it may not be so rare. Among the last 38 patients with new-onset AOSD seen at our center (Nantes), five (13%) were diagnosed in the ICU. In a recent single-center series, 19 out of 57 patients (33%) developed one or more organ complications (with or without organ failure) [3]. In the present study, we wanted to specifically focus on life-threatening AOSD, i.e. on ICU patients with overt organ failure. This stringent inclusion criterion explains the rather small size of this multicenter nationwide series.

Both in our series and in published cases, we found that three key AOSD organ manifestations can lead to ICU admission. The most frequent is respiratory failure due to lung damage and/or pleural effusion. The second is cardiocirculatory failure (non-cardiogenic shock, myocarditis, and/or cardiac tamponade). The third complication is hematologic disorders, including RHS and/or DIC. The majority of patients exhibit a combination of organ manifestations and one-third has MOF. These complications occurred mostly during the first flare. Expectedly, patients had usual signs of AOSD. Half of them exhibited the classic triad of high-grade fever with evanescent maculopapular rash and arthromyalgia [2]. Other clues to the diagnosis, including sore throat, serositis, neutrophil leukocytosis, and hepatic cytolysis were highly prevalent. However, patients suffered significant diagnostic delay. Median time between fever onset and treatment was 3 weeks. Even once organ failure had occurred, therapeutic delay remained significant. The main issue was the difficulty to abandon the sepsis hypothesis, as reflected by the amount of antibiotics these patients received before AOSD diagnosis. High-grade fever, sore throat, lung infiltrates, hepatic cytolysis, and neutrophilic leukocytosis clearly misguided clinicians, along with the negativity of autoimmunity screening.

RHS is the most frequent AOSD complication [3, 17–19]. Both entities share pathophysiologic pathways and clinicobiological features [2, 17–19, 26–30]. As for RHS, hyperferritinemia is a useful diagnostic clue for AOSD [2, 21, 22]. The sensitivity and specificity of > 1000 ng/ml hyperferritinemia are 67–80% and 36–41%, respectively, outside the ICU. In our series, all patients had > 2000 ng/mL and 17/20 (85%) reached 10,000 ng/ml. Two out of three patients without such a massive hyperferritinemia only suffered from cardiac tamponade. In recent AOSD series, the incidence of RHS is 15%. Its mortality rate is 10 to 15% [3, 17–19], which is lower than in infection and/or malignancy-related RHS [17–19]. No validated criteria are available for the diagnosis of AOSD-related RHS. We found that biological parameters that best correlated with bone marrow hemophagocytosis were thrombocytopenia and hepatic cytolysis. Paradoxically, other cytopenias and ferritin level had less predictive value, which emphasis the role of macrophage activation in AOSD, beyond hemophagocytosis. Recently, Bae et al. studied 109 AOSD patients including 21 with clinically defined RHS [18]. They found that low platelet count, anemia, and hepatomegaly were significant predictors of clinically defined RHS, but that leukocyte count and ferritin level were not. A scoring system has recently been proposed to estimate the probability of RHS (H-score) of any cause [26]. Missing data precluded its calculation in our patients. Interestingly, this has been found to predict survival in an Asian AOSD cohort [27].

AOSD, RHS, and sepsis share pathophysiological aspects, including the prominent role of pro-inflammatory cytokines IL-1, IL-6, and IL-18 [2, 28–33]. Interestingly, a recent study has shown that clinicobiological assessment is more reliable than cytokine profiling to distinguish AOSD from sepsis [33]. As for RHS, a careful analysis of patients’ biological profile is an important step to suspect AOSD [2, 32]. In the appropriate clinical setting, the combination of absolute or relative thrombocytopenia or hypofibrinogenemia, and massive hyperferritinemia should lead to consider the diagnosis of AOSD.

Our second objective was to assess the prognosis and response to therapy. Two patients of ours succumbed to AOSD (10%). Yet, mortality may be higher since some patients with fatal AOSD have certainly been missed. A significant proportion of patients experienced classical ICU complications and/or sequelae such as distal necrosis or neuropathy, but long-term prognosis was favorable.

Overall, only half the patients responded to CS. We found that these patients tend to have a less severe disease, and lower ferritin levels. IVIgs were frequently used as second-line therapy in patients who failed CS. Indeed, persisting or recurrent fever under CS in an ICU patient raises the issue of superimposed infection. In such circumstances IVIgs are an appealing therapy even if little data are available regarding their use in AOSD. Our study and literature data show that IVIgs are rarely effective, with an overall response rate of only 41%. By contrast, we found that IL-1-targeting therapy with anakinra was highly effective. In our series, five patients (25%) received anakinra in the ICU, with a response rate of 80%. One of the limitations of its use is the lack of data for patients with severe renal failure. Indeed, the only patient that did not fulfill our response criteria (i.e leaving ICU without further treatment) died after anakinra was stopped due to persistent fever, infections, and AKI requiring RRT. Twenty years ago, a phase III randomized controlled trial investigated the efficacy of anakinra in ICU patients with severe sepsis [34]. In this trial, stopped for futility, 116 patients had received anakinra. Mortality rate was not different from the placebo group. Of note, a recently published post hoc analysis suggests that anakinra could be beneficial in sepsis patients with features of RHS [35]. A significant proportion of our patients had persistent disease activity after ICU discharge. At 3 months, 16 out of 18 required additional treatment. At this time point, 11 patients had received anakinra, which was effective in ten cases (91%).

Our series have several limitations owing to its retrospective nature, limited size, and heterogeneity. However, retrospective studies are essential tools for the study of such rare diseases. They can also provide meaningful information regarding the efficacy and safety of therapeutic strategies in complex yet real-life situations.

## Conclusions

AOSD should be recognized as a potential sepsis mimic in patients with fever of unknown origin admitted to ICU. In the absence of diagnostic gold standard, intensivists should be aware of the clinical and biological clue to this diagnosis. Fifty percent of patients are refractory to CS, thus early intensive treatment may be discussed on an individual patient basis [36]. Whether life-threatening AOSD is a distinct subset or merely the result of diagnostic delay remains to be determined. Further studies are needed to identify risk factors for life-threatening complications in patients with AOSD that had not yet entered the ICU.

## Additional files

Additional file 1: Flow chart of life-threatening adult-onset Still disease. (PDF 363 kb)

Additional file 2: Additional information about the manuscript methods and literature review references are provided. (DOCX 27 kb)

Additional file 3: Comparison of patients according to response to corticosteroids in the ICU. (DOCX 14 kb)

Additional file 4: Treatment of 20 AOSD in the ICU: treatments, timing and outcome. (PDF 510 kb)

Additional file 5: Example of two patients treated by anakinra. (PDF 265 kb)

## Abbreviations

ADAMTS 13: A disintegrin and metalloprotease with thrombospondin type I repeats-13
AKI: acute kidney injury
AOSD: Adult-onset Still disease
ARDS: Acute respiratory distress syndrome
ARF: Acute respiratory distress
ASAT: Aspartate aminotransferase
CRP: C-reactive protein
CS: Corticosteroids
DIC: Disseminated intravascular coagulation
ICU: Intensive care unit
IL: interleukin
IVIgs: Intravenous immunoglobulins
MOF: Multiple organ failure
PA: Prothrombin activity
RHS: Reactive hemophagocytic syndrome
RRT: Renal replacement therapy
SAPS II: Simplified Acute Physiology Score II
TMA: Thrombotic microangiopathy
TNF-α: tumor necrosis factor alpha
