# Supplementary material for: Diagnostic and management of life-threatening Adult-Onset Still Disease: a French nationwide multicenter study and systematic literature review
Source: Crit Care. 2018 Apr 11;22:88. doi: 10.1186/s13054-018-2012-2 (PMC5896069; doi:10.1186/s13054-018-2012-2)
Supplement: Supplementary file 1 — Flow chart of life-threatening adult-onset Still disease. (PDF 363 kb) [file 13054_2018_2012_MOESM1_ESM.pdf]

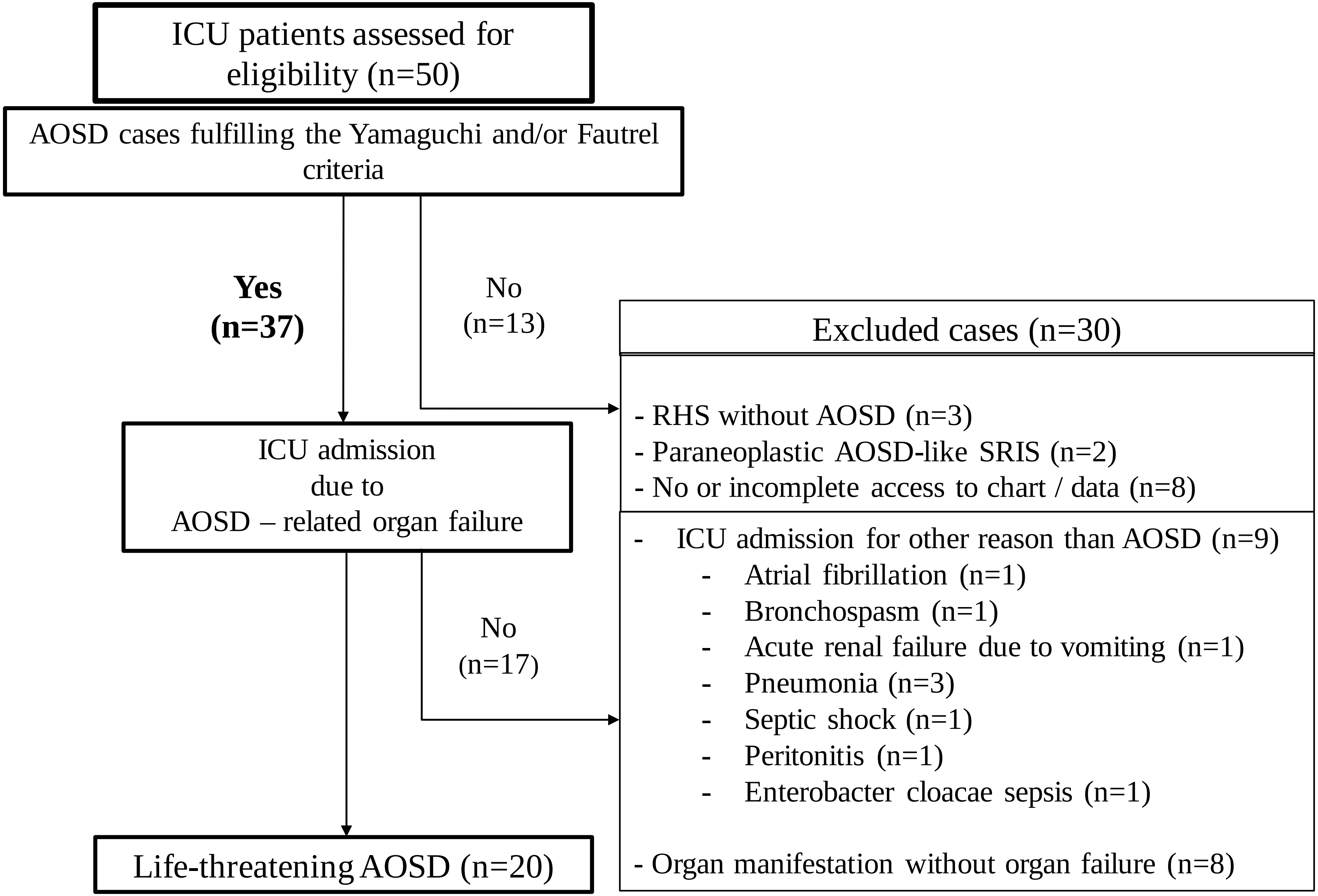

Flow chart of life-threatening adult onset still disease

AOSD : adult onset still disease ; ICU : intensive care unit
