# Supplementary material for: Diagnostic and management of life-threatening Adult-Onset Still Disease: a French nationwide multicenter study and systematic literature review
Source: Crit Care. 2018 Apr 11;22:88. doi: 10.1186/s13054-018-2012-2 (PMC5896069; doi:10.1186/s13054-018-2012-2)
Supplement: Supplementary file 2 — Additional information about the manuscript methods and literature review references are provided. (DOCX 27 kb) [file 13054_2018_2012_MOESM2_ESM.docx]

**Additional file 2**

**Additional Methods**

**Case Series : Definition of organ manifestations and Organ failure**

Respiratory failure was defined as the requirement for > 50% FiO2 oxygen supply and/or mechanical ventilation, Cardio-circulatory failure was defined as systolic blood pressure < 100 mmHg that required pressor amines administration for more than 12 hours. Acute kidney injury (AKI) was defined as doubling of serum creatinine that persisted > 24h after hemodynamic and fluid resuscitation. Disseminated intravascular coagulation (DIC) was defined as the combination of thrombocytopenia, prolonged prothrombin activity, and elevated fibrin-related markers. Hemophagocytosis was defined as the observation of macrophages engulfing erythrocytes, platelets, leukocytes, or precursor cells.on bone marrow examination. Neurologic dysfunction was defined as altered mental status or seizure. Liver enzyme elevation was defined as a ASAT or ALAT level 3 times the upper limit of normal (ULN). Severe hepatitis was defined as the combination of persistent hepatic cytolysis > 50 ULN with factor V<50%. MOF was defined as the occurrence of organ failure in 3 or more organ systems. In patients with mixed phenotype, organ failure classification issues were solved by consensus (AW, AN, CB).

**Systematic literature review: Search strategy**

For the Pubmed search, the following MeSH terms and text words were used: adult onset still disease, adult onset still’s disease, adult-onset still disease, adult-onset still’s disease, AOSD ; associated with the following: intensive care unit, thrombotic microangiopathy, thrombotic thrombocytopenic purpura, coagulopathy, hemophagocytic lymphohistiocytosis, reactive hemophagocytic syndrome, macrophage activation syndrome, disseminated intravascular coagulation, multiple organ dysfonctions, multiple organ failure, multi-organ failure, multi organ failure, acute kidney injury, renal failure, neurological involvement, cerebral oedema, epilepticus, neurological manifestations, shock, severe systemic inflammatory response, respiratory distress, acute distress respiratory syndrome, ARDS, respiratory failure, myocardial dysfunction, myocarditis, heart failure, tamponade, pericarditis, hepatic failure, liver failure, hepatic insufficiency, hepatitis, liver transplantation.

References of all articles were also checked for cases not identified in the initial search. Only cases with well-documented clinical summaries and relevant information were included.

**Literature review references**

Acosta A, Thierer J, Conde D, et al: Acute heart failure as a form of relapse in a patient with adult-onset Still disease. *Am J Emerg Med* 2014; 32:1151.e5-6.

Ames PRJ, Walker E, Aw D, et al: Multi-organ failure in adult onset Still’s disease: a septic disguise. *Clin Rheumatol* 2009; 28 Suppl 1:S3–6.

Arlet J-B, Le THD, Marinho A, et al: Reactive haemophagocytic syndrome in adult-onset Still’s disease: a report of six patients and a review of the literature. *Ann Rheum Dis* 2006; 65:1596–1601.

Ben Ghorbel I, Lamloum M, Miled M, et al: [Adult-onset Still’s disease revealed by a pericardial tamponade: report of two cases]. *Rev Med Int* 2006; 27:546–549.

Bennett AN, Peterson P, Sangle S, et al: Adult onset Still’s disease and collapsing glomerulopathy: successful treatment with intravenous immunoglobulins and mycophenolate mofetil. *Rheumatology (Oxford)* 2004; 43:795–799.

Biron C, Chambellan A, Agard C, et al: Acute respiratory failure revealing adult-onset Still’s disease: diagnostic value of low glycosylated ferritin level. *Clin Rheumatol* 2006; 25:766–768.

Bürgi U, Mendez A, Hasler P, Hüllstrung HD: Hemophagocytic syndrome in adult-onset Still’s disease (AOSD): a must for biologics?--Case report and brief review of the literature. *Rheumatol Int* 2012; 32:3269–3272.

Buss SJ, Wolf D, Mereles D, et al: A rare case of reversible constrictive pericarditis with severe pericardial thickening in a patient with adult onset Still’s disease. *Int J Cardiol* 2010; 144:e23–25.

Carron PL, Surcin S, Plane P, et al: [Adult-onset Still’s disease, a rare cause of acute respiratory distress]. *Rev Med Int* 2000; 21:1133–1134.

Cavallasca JA, Vigliano CA, Perandones CE, Tate GA: Myocarditis as a form of relapse in two patients with adult Still’s disease. *Rheumatol Int* 2010; 30:1095–1097.

Colina M, Govoni M, Trotta F: Fatal myocarditis in adult-onset Still disease with diffuse intravascular coagulation. *Rheumatol Int* 2009; 29:1355–1357.

B Dua A, M Manadan A, P Case J: Adult Onset Still’s Disease Presenting with Acute Respiratory Distress Syndrome: Case Report and Review of the Literature. *Open Rheumatol* 2013; J 7:125–128.

Duburcq T, Delannoy P-Y, Sivova N, Leroy O: [Adult onset Still’s disease revealed by a myocarditis]. Ann Fr *Anesth Reanim* 2013; 32:50–52.

Eardley KS, Raza K, Adu D, Situnayake RD: Gold treatment, nephrotic syndrome, and multi-organ failure in a patient with adult onset Still’s disease. *Ann Rheum Dis* 2001; 60:4–5.

El Karoui K, Karras A, Lebrun G, et al: Thrombotic microangiopathy and purtscher-like retinopathy associated with adult-onset Still’s disease: a role for glomerular vascular endothelial growth factor? *Arthritis Rheum* 2009; 61:1609–1613.

Fujii K, Kitamura Y, Osugi Y, et al: A case of adult-onset Still’s disease complicated by hemophagocytic syndrome and interstitial pneumonia with pneumomediastinum/recurrent pneumothorax. *Int J Rheum Dis* 2012; 15:e60–62.

Fukaya S, Yasuda S, Hashimoto T, et al: Clinical features of haemophagocytic syndrome in patients with systemic autoimmune diseases: analysis of 30 cases. *Rheumatology (Oxford)* 2008; 47:1686–1691.

Gianella S, Schaer DJ, Schwarz U, et al: Retinal microangiopathy and rapidly fatal cerebral edema in a patient with adult-onset Still’s disease and concurrent macrophage activation syndrome. *Am J Hematol* 2008; 83:424–427.

Gopal M, Cohn CD, McEntire MR, Alperin JB: Thrombotic thrombocytopenic purpura and adult onset Still’s disease. *Am J Med Sci* 2009; 337:373–376.

Hamidou M, Boutoille D, Masseau A, et al: [Adult-onset Still disease with hemophagocytic syndrome treated with cyclosporine]. *Presse Med* 2005; 34:1634–1636.

Harmanci Ö, Kav T, Sökmensüer C, et al: Successful management of severe acute liver disease related with adult-onset Still’s disease in a pregnant patient. *Turk J Gastroenterol* 2013; 24:61–64.

Hong YH, Lee CK: A case of adult onset Still’s disease with systemic inflammatory response syndrome complicated by fatal status epilepticus. *Rheumatol Int* 2008; 28:931–933.

Hot A, Toh M-L, Coppéré B, et al: Reactive hemophagocytic syndrome in adult-onset Still disease: clinical features and long-term outcome: a case-control study of 8 patients. *Medicine (Baltimore)* 2010; 89:37–46.

Izzedine H, Brocheriou I, Martinez V, et al: Fever, nephrotic syndrome, and rapidly progressive renal failure. *Kidney Int* 2007; 72:651–656.

Jacquet-Lagrèze M, Hautin E, Floccard B, et al: [Cardiac tamponade revealing an adult onset Still’s disease]. *Ann Fr Anesth Reanim* 2013; 32:65.

Jadhav P, Nanayakkara N: Myocarditis in adult onset Stills disease. *Int J Rheum Dis* 2009; 12:272–274.

Kato T, Fujii K, Wakabayashi T, et al: A case of cutaneous polyarteritis nodosa manifested by spiking high fever, arthralgia and macular eruption like adult-onset Still’s disease. *Clin Rheumatol* 2006; 25:419–421.

Kobayashi M, Takahashi Y, Yamashita H, et al: Benefit and a possible risk of tocilizumab therapy for adult-onset Still’s disease accompanied by macrophage-activation syndrome. *Mod Rheumatol* 2011; 21:92–96.

Kuek A, Weerakoon A, Ahmed K, Ostör AJK: Adult-onset Still’s disease and myocarditis: successful treatment with intravenous immunoglobulin and maintenance of remission with etanercept. *Rheumatology (Oxford) 2007*; 46:1043–1044.

Lee W-S, Yoo W-H: Rituximab for refractory adult-onset Still’s disease with thrombotic microangiopathy. *Rheumatology (Oxford)* 2014; 53:1717–1718.

Loh NK, Lucas M, Fernandez S, Prentice D: Successful treatment of macrophage activation syndrome complicating adult Still disease with anakinra. *Intern Med J* 2012; 42:1358–1362.

Maeshima K, Ishii K, Iwakura M, et al: Adult-onset Still’s disease with macrophage activation syndrome successfully treated with a combination of methotrexate and etanercept. *Mod Rheumatol* 2012; 22:137–141.

Manganelli P, Fietta P, Zuccoli P: Adult-onset Still’s disease with respiratory distress syndrome, polyserositis and disseminated intravascular coagulation: a case with a fatal outcome. *Clin Exp Rheumatol* 2003; 21:139.

Masuyama A, Kobayashi H, Kobayashi Y, et al: A case of adult-onset Still’s disease complicated by thrombotic thrombocytopenic purpura with retinal microangiopathy and rapidly fatal cerebral edema. *Mod Rheumatol* 2013; 23:379–385.

Matsumoto K, Nagashima T, Takatori S, et al: Glucocorticoid and cyclosporine refractory adult onset Still’s disease successfully treated with tocilizumab. *Clin Rheumatol* 2009; 28:485–487.

Mejjad O, Vittecoq O, Tamion F, et al: A shock associated with adult-onset Still’s disease. *Joint Bone Spine* 2001; 68:76–78.

Namas R, Nannapaneni N, Venkatram M, et al: An Unusual Case of Adult-Onset Still’s Disease with Hemophagocytic Syndrome, Necrotic Leukoencephalopathy and Disseminated Intravascular Coagulation. *Case Rep Rheumatol* 2014; 2014:128623.

Nishida T, Suzuki K, Kuwada N, et al: Hemophagocytic syndrome and adult Still’s disease associated with meningoencephalitis and unconsciousness. *Intern Med* 2001; 40:1037–1040.

Nishimagi E, Hirata S, Kawaguchi Y, et al: Myocardial dysfunction in a patient with adult-onset Still’s disease (AOSD). *Clin Exp Rheumatol* 2004; 22:506–507.

Okwuosa TM, Lee EW, Starosta M, et al: Purtscher-like retinopathy in a patient with adult-onset Still’s disease and concurrent thrombotic thrombocytopenic purpura. *Arthritis Rheum* 2007; 57:182-185.

Orr J, Bury Y, Hudson M, Masson S: Liver transplantation for acute liver failure caused by macrophage activation syndrome. *Transpl Int* 2013; 26:e105–108.

Pamuk ON, Pamuk GE, Usta U, Cakir N: Hemophagocytic syndrome in one patient with adult-onset Still’s disease. Presentation with febrile neutropenia. *Clin Rheumatol* 2007; 26:797–800.

Park J-H, Bae JH, Choi Y-S, et al: Adult-onset Still’s disease with disseminated intravascular coagulation and multiple organ dysfunctions dramatically treated with cyclosporine A. *J Korean Med Sci* 2004; 19:137–141.

Quéméneur T, Noel L-H, Kyndt X, et al: Thrombotic microangiopathy in adult Still’s disease. *Scand J Rheumatol* 2005; 34:399–403.

Rawal S, Einbinder Y, Rubin L, et al: Thrombotic microangiopathy in a patient with adult-onset Still’s disease. *Transfusion (Paris)* 2014; 54(11):2983-7.

Robert V, Eszto P, Perrotez J-L, et al: [Treatment by plasmapheresis of a thrombotic thrombocytopenic purpura associated to a Still’s disease: a case report]. *Ann Fr Anesth Reanim* 2006; 25:532–534.

Salamon L, Salamon T, Morovic-Vergles J: Thrombotic microangiopathy in adult-onset Still’s disease: case report and review of the literature. *Wien Klin Wochenschr* 2009; 121:583–588.

Sari I, Birlik M, Binicier O, et al: A case of adult-onset Still’s disease complicated with diffuse alveolar hemorrhage. *J Korean Med Sci* 2009; 24:155–157.

Sayarlioglu M, Sayarlioglu H, Ozkaya M, et al: Thrombotic thrombocytopenic purpura-hemolytic uremic syndrome and adult onset Still’s disease: case report and review of the literature. *Mod Rheumatol* 2008; 18:403–406.

Singh B, Biboa J, Musuku S, et al: Reversal of severe hepatitis with infliximab in adult-onset Still’s disease. *Am J Med* 2013; 126:e3–4.

Slovis BS, Eyler AE: A 33-year-old man with pharyngitis, transient rash, and multiorgan system failure. *Chest* 2007 132:1080–1083.

Stoica GS, Cohen RI, Rossoff LJ: Adult Still’s disease and respiratory failure in a 74 year old woman. *Postgrad Med J* 2002; 78:97–98.

Suleiman M, Wolfovitz E, Boulman N, Levy Y: Adult onset Still’s disease as a cause of ARDS and acute respiratory failure. *Scand J Rheumatol* 2002; 31:181–183.

Sumida K, Ubara Y, Hoshino J, et al: Etanercept-refractory adult-onset Still’s disease with thrombotic thrombocytopenic purpura successfully treated with tocilizumab. *Clin Rheumatol* 2010; 29:1191–1194.

Taccone FS, Lucidi V, Donckier V, et al: Fulminant hepatitis requiring MARS and liver transplantation in a patient with Still’s disease. *Eur J Intern Med* 2008; 19:e26–28.

Wang H-P, Chen H-A, Chen C-H, et al: Adult Still’s disease patient developed thrombotic microangiopathy with diffuse digital gangrene. *Scand J Rheumatol* 2007; 36:76–78.

Yang D-H, Chang D-M, Lai J-H, et al: Etanercept as a rescue agent in patient with adult onset Still’s disease complicated with congestive heart failure. *Rheumatol Int* 2008; 29:95–98.

Yang C-C, Lee JY-Y, Liu M-F, Ho C-L: Adult-onset Still’s disease with persistent skin eruption and fatal respiratory failure in a Taiwanese woman. *Eur J Dermatol EJD* 2006; 16:593–594.

Yeh H-M, Liu M-F, Chang K-K, et al: Adult-onset Still’s disease complicated with hemophagocytic syndrome. *J Formos Med Assoc* 2010; 109:85–88.

Yoshizaki A, Kawakami A, Aramaki T, et al: Preferential recovery by an intensive initial therapy from hemophagocytic syndrome complicated with adult onset Still;s disease presenting as agranulocytosis and hypercytokinemia. *Clin Exp Rheumatol* 2008; 26:383.

Zhang X-H, Han Y-M, Wang W-W, et al: Hemophagocytic syndrome secondary to adult-onset Still’s disease but very similar to lymphoma. *Int J Clin Exp Pathol* 2012; 5:377–381.

Zhao H, Yuan Y, Li Y, et al: Encephalic large arteries narrowness and peripheral neuropathy in a patient with adult-onset Still’s disease. *Rheumatol Int* 2008; 28:1261–1264.
