# Supplementary material for: Diagnostic and management of life-threatening Adult-Onset Still Disease: a French nationwide multicenter study and systematic literature review
Source: Crit Care. 2018 Apr 11;22:88. doi: 10.1186/s13054-018-2012-2 (PMC5896069; doi:10.1186/s13054-018-2012-2)
Supplement: Supplementary file 3 — Comparison of patients according to response to corticosteroids in the ICU. (DOCX 14 kb) [file 13054_2018_2012_MOESM3_ESM.docx]

**Additional Table**

| **Tableau 1. Comparison of patients according to response to corticosteroids in the ICU** | | | | | | |
| --- | --- | --- | --- | --- | --- | --- |
|  | | **Response to CS** | **Non Response to CS** | | **p** |  |
| **AOSD features** | | | | | | |
| Age | | | 37 (18-64) | 33 (22-51) | 0,941 | |
| Male | | | 6 (60%) | 6 (60%) | 0,465 | |
| **Laboratory** | | | | | | |
| Leukocytes (x10^9^/mL) | | | 26 (12-44) | 20,5 (0-31) | 0,733 | |
| Hemoglobin (g/dL) | | | 8 (6-13) | 10 (6-12) | 0,424 | |
| Platelet (x10^9^/mL) | | | 347 (39-638) | 93 (42-434) | 0,286 | |
| PA (%) | | | 62,5 (40-87) | 50 (30-90) | 0,560 | |
| ASAT (UI/L) | | | 91 (31-2963) | 202 (48-857) | 0,213 | |
| Creatinine (µmol/L) | | | 89 (41-334) | 165 (36-580) | 0,351 | |
| CRP (mg/L) | | | 315 (152-493) | 363 (100-495) | 0,406 | |
| Serum Ferritin (ng/mL) | | | 6531 (655-38 000) | 33 022 (14 178-147 568) | 0,017 | |
| **Organ manifestations** | | | | | | |
| SAPS II | | 22 (16-50) | | 37,5 (17-88) | 0,27 | |
| Hematologic failure | | 4 (40%) | | 9 (90%) | 0,057 | |
| Shock | | 5 (50%) | | 5 (50%) | 0,500 | |
| Respiratory Distress | | 6 (60%) | | 9 (90%) | 0,396 | |
| Acute Kidney Injury | | 2 (20%) | | 5 (50%) | 0,272 | |
| Multiple organ failure | | 2 (20%) | | 6 (60%) | 0,157 | |
| *ASAT : aspartate aminotransferase ; ALAT : alanine aminotransferase ; CRP : C reactive protein ; CS NR : corticosteroids no responder ; CS R : corticosteroids responder ; SAPS II : Simplified Acute Physiology Score II ; PA : prothrombin activity  Frequency (%) ; Median (min-max)* | | | | | | |
