# Supplementary material for: Diagnostic and management of life-threatening Adult-Onset Still Disease: a French nationwide multicenter study and systematic literature review
Source: Crit Care. 2018 Apr 11;22:88. doi: 10.1186/s13054-018-2012-2 (PMC5896069; doi:10.1186/s13054-018-2012-2)
Supplement: Supplementary file 4 — Treatment of 20 AOSD in the ICU: treatments, timing and outcome. (PDF 510 kb) [file 13054_2018_2012_MOESM4_ESM.pdf]

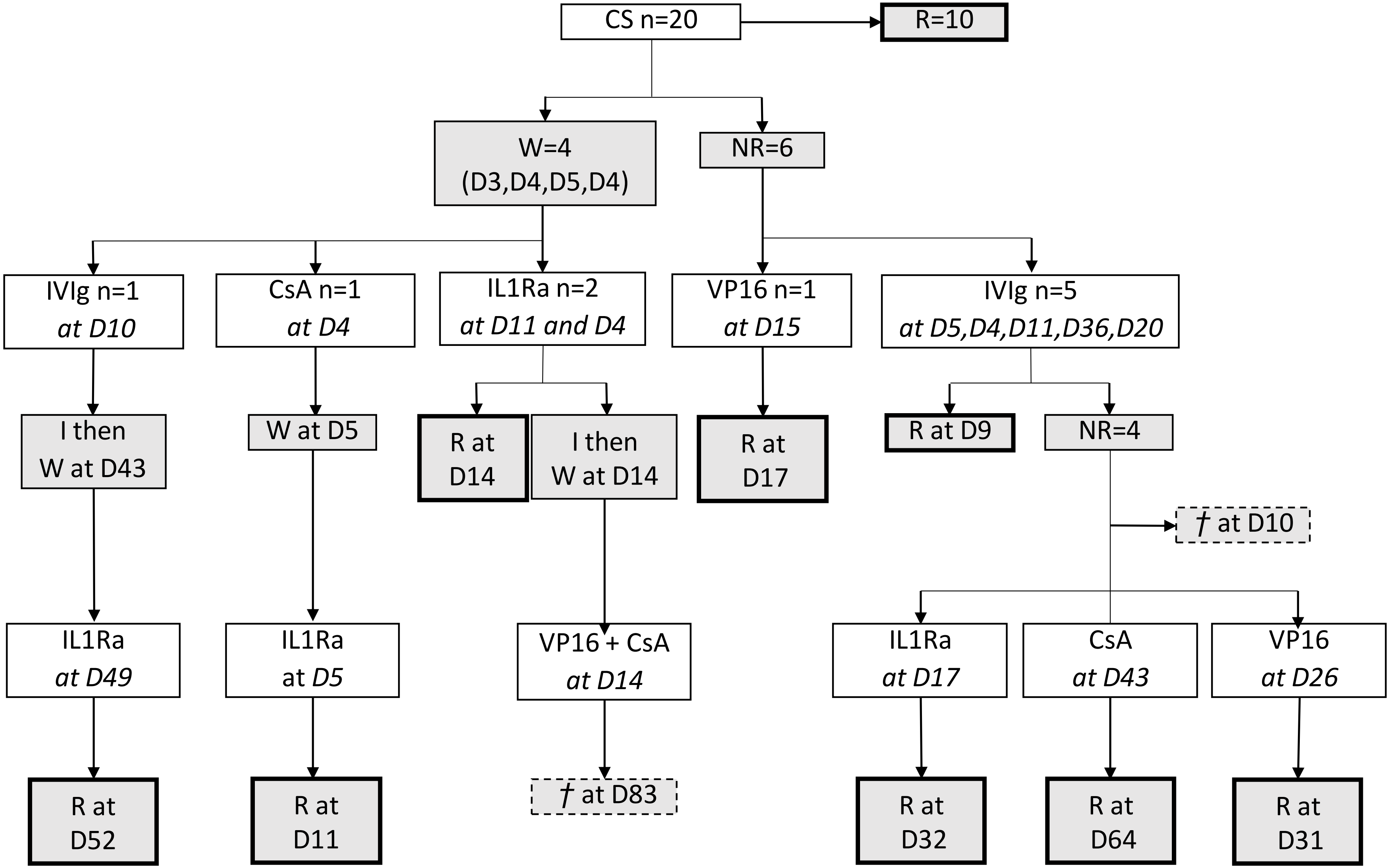

Treatment of 20 AOSD in the ICU : treatments, timing and outcome.
CS : corticosteroids ; CsA : ciclosporin ; D : day ; I : improvement ;
IVIgs : intravenous immunoglobulins ; NR : no response ; R : response (ICU discharge) ; VP16 : etoposide ; W : worsening ; † : death.
