# Supplementary material for: Diagnostic and management of life-threatening Adult-Onset Still Disease: a French nationwide multicenter study and systematic literature review
Source: Crit Care. 2018 Apr 11;22:88. doi: 10.1186/s13054-018-2012-2 (PMC5896069; doi:10.1186/s13054-018-2012-2)
Supplement: Supplementary file 5 — Example of two patients treated by anakinra. (PDF 265 kb) [file 13054_2018_2012_MOESM5_ESM.pdf]

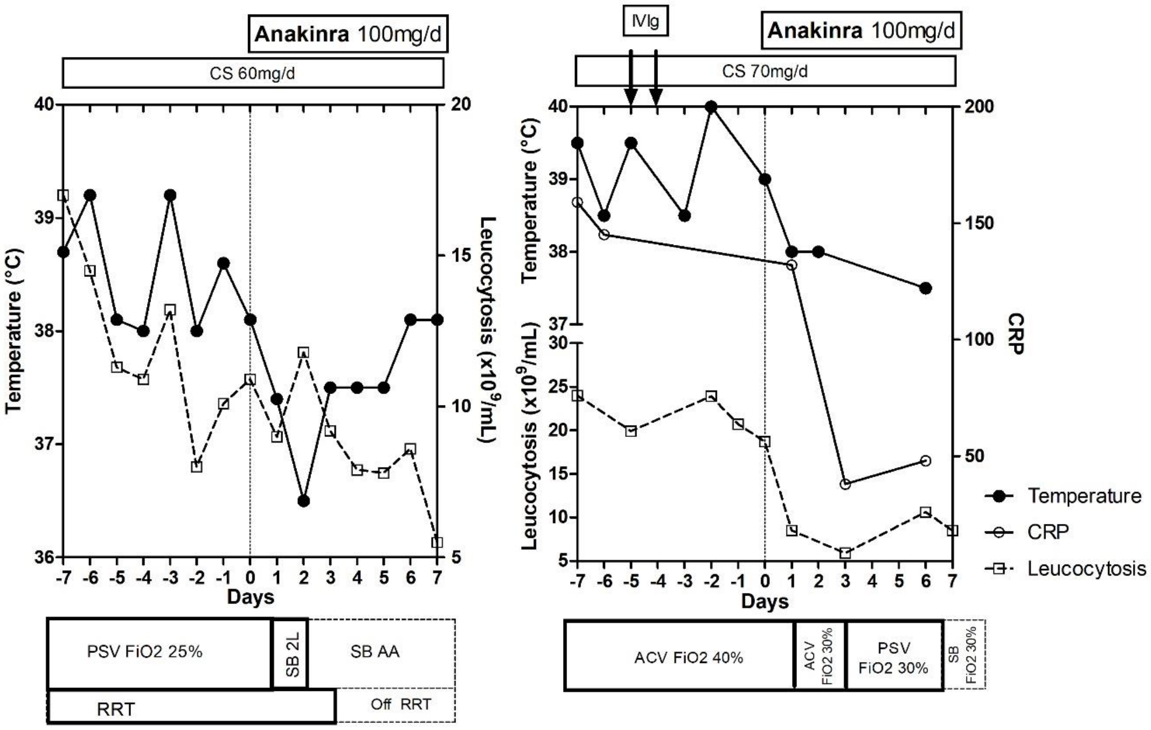

Example of 2 patients treated by anakinra.

ACV : assisted controlled ventilation ; CRP : C reactive protein ; CS : corticosteroids ; IVIgs : intravenous immunoglobulins ; PSV : pressure support ventilation ; RRT : renal replacement therapy ; SB : spontaneous breathing
